# Experimental quantum imaging distillation with undetected light

**DOI:** 10.1126/sciadv.adg9573

**Published:** 2023-08-30

**Authors:** Jorge Fuenzalida, Marta Gilaberte Basset, Sebastian Töpfer, Juan P. Torres, Markus Gräfe

**Affiliations:** ^1^ Fraunhofer Institute for Applied Optics and Precision Engineering IOF, Albert-Einstein-Str. 7, 07745 Jena, Germany.; ^2^ Institute of Applied Physics, Technical University of Darmstadt, Schloßgartenstraße 7, 64289 Darmstadt, Germany.; ^3^ Friedrich Schiller University Jena, Abbe Center of Photonics, Albert-Einstein-Str. 6, 07745 Jena, Germany.; ^4^ ICFO-Institut de Ciencies Fotoniques, The Barcelona Institute of Science and Technology, 08860 Castelldefels, Spain.; ^5^ Department of Signal Theory and Communications, Universitat Politecnica de Catalunya, 08034 Barcelona, Spain.

## Abstract

Imaging based on the induced coherence effect makes use of photon pairs to obtain information of an object without detecting the light that probes it. While one photon illuminates the object, only its partner is detected, so no measurement of coincidence events is needed. The sought-after object’s information is revealed, observing a certain interference pattern on the detected photon. Here, we demonstrate experimentally that this imaging technique can be made resilient to noise. We introduce an imaging distillation approach based on the interferometric modulation of the signal of interest. We show that our scheme can generate a high-quality image of an object even against noise levels up to 250 times the actual signal of interest. We also include a detailed theoretical explanation of our findings.

## Teaser

Phase-shifting digital holography enables quantum imaging distillation with undetected light.

## INTRODUCTION

Quantum imaging (*1*) is an emerging and promising field in quantum technologies with certified advantages over classical protocols. This has been demonstrated in different scenarios: in schemes that work in the low-photon flux regime (*2*, *3*), in schemes that make use of undetected probing photons (*4*, *5*), for superresolution imaging (*6*–*9*), sub–shot noise imaging (*10*–*12*), or enhanced two-photon absorption processes (*13*). Moreover, protocols in quantum imaging with no classical counterpart have been developed on the basis of quantum interference (*14*) and entanglement (*15*, *16*). In recent years, it has also been proven that quantum imaging protocols can be resilient to noise (*17*–*19*).

Distillation (also known as purification) is the process wherein the decoherence introduced in a quantum system by the environment can be removed (*20*). In quantum imaging, the effect of the environment can be modeled through classical illumination superimposed over a quantum image on the camera. Because most cameras only detect intensity, quantum and classical images seem to be indistinguishable. However, quantum correlations of photon pairs can be used to differentiate the quantum image from a classical one. Quantum imaging distillation has been implemented with one and several photon pair degrees of freedom (*21*–*26*). To the best of our knowledge, every implementation, to date, has used the joint detection of photon pairs. In this work, we introduce and experimentally verify a quantum imaging distillation technique that uses the detection of single photons only.

Quantum imaging with undetected light (QIUL) (*4*, *27*–*29*) is a two-photon wide-field interferometric imaging technique. In QIUL, one photon illuminates an object, and its partner photon is detected on the camera. The photon that illuminates the object remains undetected. Using an interferometric configuration, the object information is transferred to the detected photon interference pattern. Because of its unique detection advantage, QIUL has been used to probe samples with unconventional wavelengths while visible light is detected (*30*–*34*). Up to date, the effects of noise in QIUL have not yet been explored.

Here, we introduce a source of noise in a QIUL scheme and study the resilience of the quantum imaging technique. The properties of the noise, its intensity and variance, are changed during this study. We perform a quantum imaging distillation technique based on quantum phase-shifting digital holography (*35*). Our distillation technique uses phase modulation of the undetected photon to vary the interference pattern detected on the camera. We notice that, if the intensity difference of the interference patterns is bigger than the variance of the noise, then the quantum image can be distilled. We also observe that the noise variance affects the distilled quantum images linearly in their phase estimation. Our technique shows a good performance, even for noise intensities above 250 times the quantum signal intensity.

## RESULTS

### Distillation principle

**Fig. 1.**
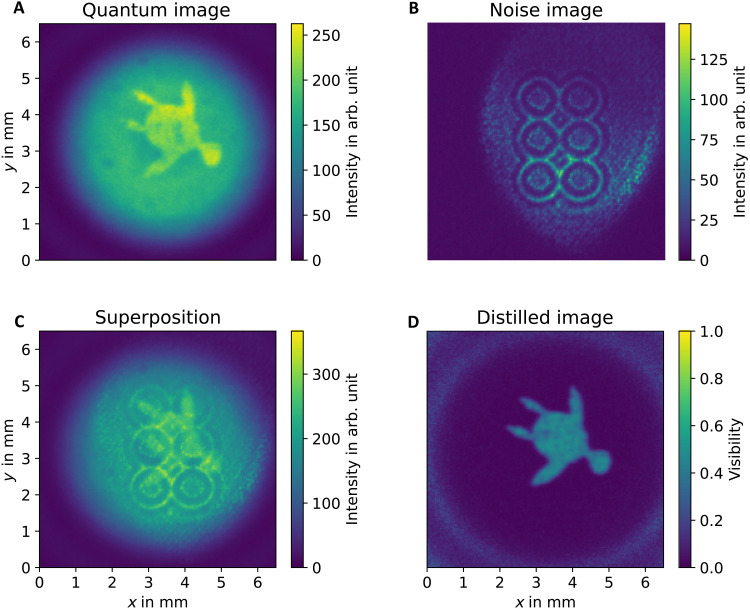
Principle of quantum imaging distillation. We use a quantum imaging distillation protocol to remove a noisy image from a quantum image. (**A**) The quantum image that we aim to distill. (**B**) The noise image that is superimposed on the quantum image. (**C**) The superposition of noise and quantum images. (**D**) The resulting distilled image from noise.

Quantum imaging distillation is a process whereby a quantum image is cleaned from noise. To explain our distillation technique, let us consider two images: a quantum image, which is acquired by illuminating the sample with nonclassical light, and a noise image, which is an image detected at the camera and generated with classical illumination. These two images are shown in Fig. 1 (A and B, respectively). A noise image is an unwanted signal that is superimposed over a quantum image on the camera. This image superposition is shown in Fig. 1C. To distill an image, different photon pair degrees of freedom can be used, e.g., frequency, time, or spatial correlations. We use the amplitude modulation of quantum holography with undetected light (QHUL) (*35*), which is an interferometric quantum imaging technique (*4*). In QHUL, the object information is carried into a single-photon interference pattern. When the noise reaches the camera, if the intensity difference of QHUL is bigger than the intensity variance of the noise, then the quantum image can be distilled. The resulting distilled image is shown in Fig. 1D.

### Theory

Spontaneous parametric down-conversion (SPDC) (*36*, *37*) is a well-known nonlinear process that generates photon pairs (signal and idler) mediated by the interaction of an intense pump beam with the atoms of a nonlinear crystal (*38*). Our imaging scheme makes use on a SU(1,1) interferometer, wherein a pair of signal-idler photons can be generated in one of the two propagation modes: forward and backward. The probability to generate paired photons in both modes (forward or backward) simultaneously is negligible (*39*, *40*). In the forward propagation mode, the pump, signal, and idler beams are spatially separated and, later, back-reflected into the nonlinear crystal with the help of 4f systems. Before back reflection, the idler photon is reflected from an object, with complex reflectivity *R* = ∣*R*∣exp(*i*ϕ*_R_*), placed in front of its end-mirror. In the backward propagation mode, the idler photon does not interact with the sample. The signal photons are collected by a camera, and the idler photon remains undetected.

The mean value of signal photons detected in time *T_D_* at one pixel of the camera of area *S_D_* (see the Supplementary Materials for details) is

$${\left\langle N_{S}\right\rangle }_{\delta }=2S_{0}[1+|R|\gamma \;\cos(\delta +\varphi_{R})]$$ (1)

δ is an interferometric spatially invariant phase and *S*_0_ is the number of signal photons generated in single-pass SPDC (in a time window *T_D_* and area *S_D_*). The parameter γ is related to the effective bandwidth of the signal-idler photon pairs, determined essentially by the bandwidth of the filters located in front of the camera (*35*). From Eq. 1, we see that ⟨*N_S_*⟩ changes when δ is varied. In particular, using δ = 0, π/2, π, and 3π/2, the object’s information can be retrieved by means of QHUL as follows

$$|R|=2\times \frac{{\left({\left[{\left\langle N_{S}\right\rangle }_{3\pi /2}-{\left\langle N_{S}\right\rangle }_{\pi /2}\right]}^{2}+{\left[{\left\langle N_{S}\right\rangle }_{0}-{\left\langle N_{S}\right\rangle }_{\pi }\right]}^{2}\right)}^{1/2}}{{\left\langle N_{S}\right\rangle }_{0}+{\left\langle N_{S}\right\rangle }_{\pi /2}+{\left\langle N_{S}\right\rangle }_{\pi }+{\left\langle N_{S}\right\rangle }_{3\pi /2}}$$ (2)

$$\varphi_{R}=\arctan\left(\frac{{\left\langle N_{S}\right\rangle }_{3\pi /2}-{\left\langle N_{S}\right\rangle }_{\pi /2}}{{\left\langle N_{S}\right\rangle }_{0}-{\left\langle N_{S}\right\rangle }_{\pi }}\right)$$ (3)

Equations 2 and 3 are not unique representations of ∣*R*∣ and ϕ*_R_*, and, in general, these quantities can be extracted using a different number of phases (*35*). We emphasize that, in this technique, paired photon coincidences are not needed and only signal photons are measured.

For the important case of phase estimation, we evaluate the sensitivity of QHUL obtaining the variance of ϕ*_R_* given by Eq. 3. We first calculate the variance of the signal-photon flux $\langle {\left(\text{Δ}N_{S}\right)}^{2}\rangle =\langle N_{S}^{2}\rangle -{\left\langle N_{S}\right\rangle }^{2}$. Because the coherence time *T_C_* of signal-idler photon pairs (*T_C_* ∼ 1/*B*, *B* is the effective bandwidth of SPDC) is much smaller than the detection time, we can approximate the variance of the signal-photon flux to as (see the Supplementary Materials)

$$\left\langle {\left(\text{Δ}N_{S}\right)}^{2}\rangle =\langle N_{S}\right\rangle$$ (4)

which is equivalent to considering Poissonian statistics. This result is well-known to be valid when considering a multimode signal where each mode has the same non-Poissonian statistics.

The result in Eq. 4 corresponds to the minimum signal variance achievable in QHUL. However, this variance can rapidly increase by electronic noise, e.g., camera signal-to-noise ratio, or external sources of noise, such as temperature fluctuations, airflow, and external illumination. In this work, we study the effect of an external classical illumination impinging on the camera, overlapping the quantum image of interest. Because QHUL detects only the single-photon stream of signal photons, the decoherence produced by an external source of light seems extremely harmful and, therefore, the image distillation seems highly improbable. However, we will demonstrate experimentally that the quantum imaging distillation is possible even in scenarios with considerable high levels of noise in comparison to the quantum signal intensity.

**Fig. 2.**
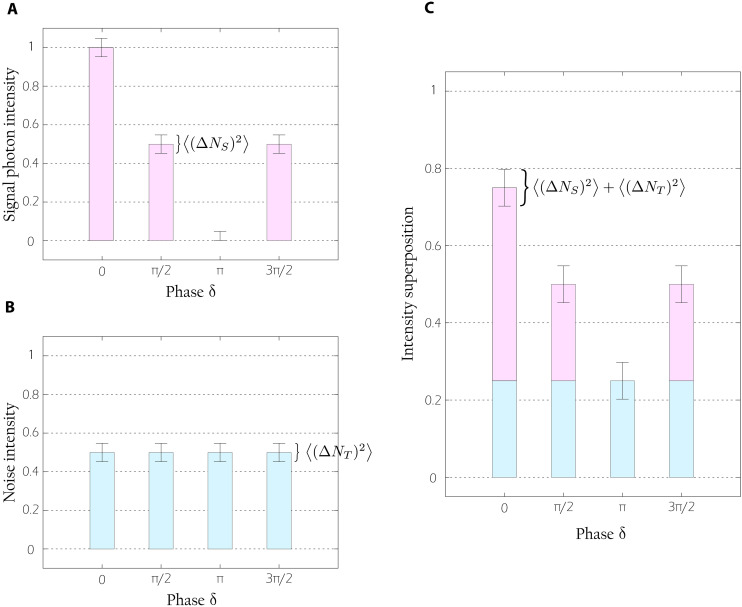
Intensity detected in one camera pixel. For visualization purposes, we have considered the same mean intensities (chart bars) and variances (error bars) for the signal photon and the noise. In (**A**), the signal photon intensity (in pink) for different values of δ is presented. This intensity fluctuation allows us to compute the object information using QHUL (*35*). In (**B**), the noise intensity (in blue) for the same phases δ is presented. In contrast to the signal intensity, the noise intensity is not affected by the value of δ. In (**C**), the noise and signal intensities are added. Because the noise intensity does not change, its contribution just sets a higher background. On top of it, signal photon intensity still changes, and the total variance is its previous variance plus the noise variance. Thus, an external source of noise increases the shot noise of QHUL.

QHUL uses interferometric modulation of the signal photon to retrieve the object information, as shown in Eqs. 2 and 3. We show that this modulation can also be used for distillation purposes, which is depicted in Fig. 2. For each value of the interference phase δ, the signal photon has a well-defined intensity and variance, given by Eqs. 1 and 4, respectively. In Fig. 2A, signal intensity (variance) is represented with pink bar charts (error bars). In contrast, the intensity of a stochastic noise ⟨*N_T_*⟩ fluctuates randomly with a variance ⟨(Δ*N_T_*)^2^⟩. For the sake of simplicity, we consider (but are not restricted to) the case where the noise has the same total mean intensity and variance than the signal photon. While the mean noise intensity is ⟨*N_T_*⟩, the mean signal intensity corresponds to {min(⟨*N_S_*⟩) + max(⟨*N_S_*⟩)}/2. In Fig. 2B, the noise intensity (variance) is represented with blue bar charts (error bars). As a result of adding these two intensities, see Fig. 2C, the background increases up to the noise intensity, while the signal intensity varies on top of it. The noise variance contributes to the signal intensity variance, i.e., the shot noise increases. In this way, one can infer that, if the difference of the signal intensity is higher than the noise variance, then the quantum image can be distilled. In addition, the shot noise of QHUL always increases if the noise intensity and/or its variance increases. More details are given in the Supplementary Materials.

Let us analyze the performance of our distillation technique by considering a generalization of Eq. 2. For QHUL with *M* phase steps, we have that

$$\varphi_{R}=-\tan^{-1}\left(\sum_{j}{\left\langle N_{S}\right\rangle }_{j}\;\sin\delta_{j}/\sum_{j}{\left\langle N_{S}\right\rangle }_{j}\;\cos\delta_{j}\right)$$ (5)

with δ*_j_* = *j* 2π/*M* and *j* = 0, 1, …, *M* − 1. The phase variance, including noise, reads

$$\langle {\left(\text{Δ}\varphi_{R}\right)}^{2}\rangle =\sum_{j}{\left(\frac{\partial \varphi_{R}}{\partial {\left\langle N_{S}\right\rangle }_{j}}\right)}^{2}\left[{\left\langle {\left(\text{Δ}N_{S}\right)}^{2}\right\rangle }_{j}+\langle {\left(\text{Δ}N_{T}\right)}^{2}\rangle \right]$$ (6)

where

$${\left(\frac{\partial \varphi_{R}}{\partial {\left\langle N_{S}\right\rangle }_{j}}\right)}^{2}=\frac{1}{M^{2}\;\gamma^{2}\;{|R|}^{2}\;S_{0}^{2}}\sin^{2}(\varphi_{R}+\delta_{j})$$ (7)

Replacing Eqs. 4 and 7 into Eq. 6 and considering *n* measurements, we obtain that the variance of phase estimation is

$$\langle {\left(\text{Δ}\varphi_{R}\right)}^{2}\rangle =\frac{1}{n\;S_{0}}\;\frac{1}{M\;V^{2}}\left\{1+\frac{\left\langle {\left(\text{Δ}N_{T}\right)}^{2}\right\rangle }{2\;S_{0}}\right\}$$ (8)

*S*_0_ is the number of idler photons that illuminate the object whose phase we want to estimate, ⟨(Δ*N_T_*)^2^⟩ is the variance of the number of background photons that illuminate the detector, and *V* = ∣*R*∣ γ is the visibility of the signal-photon flux interference pattern as a function of the phase δ. The visibility is defined by *V* = {max(⟨*N_S_*⟩) − min(⟨*N_S_*⟩)}/{max(⟨*N_S_*⟩) + min(⟨*N_S_*⟩)}. Equation 8 contains two contributions to the variance of the phase: The first term comes from the quantum illumination, and the second term comes from the noise illumination. While the former can be shown to be well described by Poissonian statistics (see the Supplementary Materials), the latter depends on the specific characteristics of the noise illumination.

### Quantum imaging

**Fig. 3.**
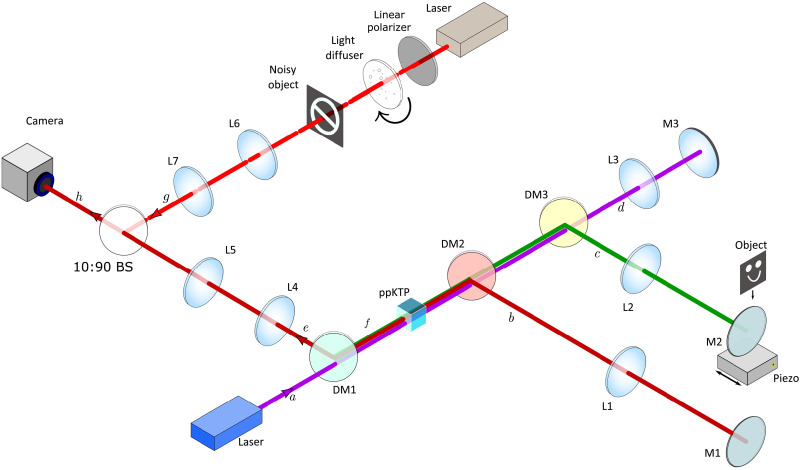
Setup. The signal and idler beams (in paths *b* and *c*, respectively) are generated by the pump beam in path *a* interacting with the nonlinear crystal [periodically poled potassium titanyl phosphate (ppKTP)] in the forward direction, while paths *e* and *f* represent propagations of the down converted beams generated after the pump beam is reflected back into the crystal by mirror M3 in path *d*. An object in path *c* is illuminated with the idler beam in the Fourier plane of the crystal. To create the noise, a laser diode of the same wavelength as the signal photon (910 nm) is used. The signal beam in path *e* is merged with the noise in path *g* before reaching the camera with a 10:90 beam splitter (BS). On the detector plane, we obtain the quantum image with lenses L2, L4, and L5, and the noise image with lenses L6 and L7. Different type of noise are created with a linear polarizer and a light diffuser in path *g*. The linear polarizer controls the pump power of the diode laser. The diffuser that consists on a rotating ground glass plate produces a speckle pattern of the noise source. The speed of the rotation is controlled through the glass plate motor interface.

A sketch of our experimental implementation is depicted in Fig. 3. For our quantum image, we used a SU(1,1) nonlinear interferometer (*41*) in a Michelson configuration, where its input/output is a nonlinear medium. Our crystal is a periodically poled potassium titanyl phosphate of 2 mm by 2 mm by 1 mm (length by width by height), which is pumped bidirectionally (*a* and *f* directions) by a continuous wave (CW) laser at 405 nm and with average power of 90 mW. Because of its strong χ^(2)^-nonlinearity, a photon pair (signal and idler photons), is generated through SPDC into the paths *a* or *f* but never simultaneously. Signal (idler) photons have a central wavelength of λ*_S_* = 910 nm (λ*_I_* = 730 nm).

In the forward propagation direction *a*, signal, idler, and pump beams are spatially separated with dichroic mirrors DM2 and DM3 into the paths *b*, *c*, and *d* and reflected back into the crystal with mirrors M1, M2, and M3. In front of mirror M2, an object with a complex amplitude *R* = ∣*R*∣exp(*i*ϕ*_R_*) is placed. In *b*, *c*, and *d*, lenses of focal length *f* = 150-mm transform transverse position (source plane) into transverse momentum (mirror plane). Therefore, in path *c*, a wave vector **k***_I_* representing a plane wave of the idler photon is focused to a point on the object (*27*, *28*). The interaction of idlers being absorbed or reflected by the object can be modeled with the help of a beam splitter (*29*). In the backward propagation *f*, signals are collected by a camera and idlers remain undetected. We ensured that, by placing a 800-nm-long pass filter and a 910 ± 1.5–nm interference filter in front of the camera. Our camera is the Prime BSI Scientific CMOS from Teledyne Photometrics with a pixel size of 6.5 μm. Signal photon’s transverse momentum is obtained with the lens L4 of focal length *f* = 100 mm performing a Fourier transform of the source plane. This plane is later imaged on camera with the lens L5 of focal length *f* = 150 mm. Thus, a wave vector **k***_S_* of the signal photon is focused to a point on the camera. If *a* and *f* propagation are perfectly aligned, then the photon pair emission (which-source) information is erased. Consequently, on the camera, an interference pattern of signal photons is observed (*42*). Moreover, the object information obtained herein by the idler photon, is transferred to the signal photon interference pattern (*4*, *5*); see Eq. 1. The interferometric phase δ is changed with a piezo placed below mirror M2. The object information is retrieved by using QHUL of 12 steps (*35*) with an acquisition time of *T_D_* = 1 s per image.

### Noise source

A CW diode laser of λ*_N_* = 910 nm and with a variable pump power is used to introduce noise in the system. The laser illuminates an object, which is imaged on the camera with a 4f system using the lenses L6 and L7 of focal lengths *f* = 150 mm and *f* = 125 mm, respectively. This classical image is superimposed on top of the quantum image on the camera using a 10:90 beam splitter; see Fig. 3. Properties of classical illumination, intensity and variance, are changed to evaluate the effects of noise in QHUL and our distillation performance. Experimental details about the noise properties can be found in the Supplementary Materials.

### Distillation performance to different noise intensities

**Fig. 4.**
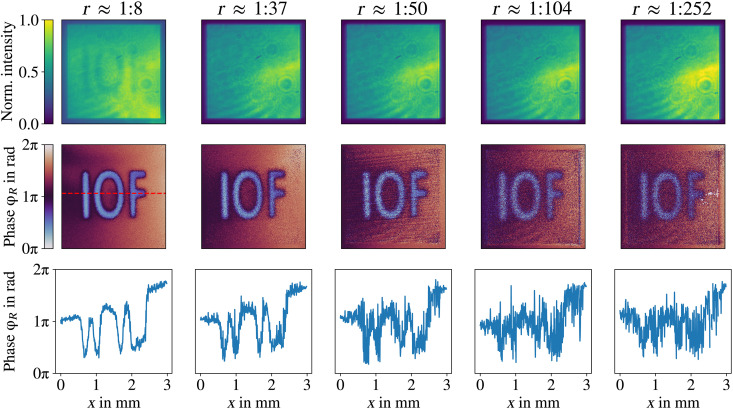
Resilience to different noise intensities. In the top row, the superpositions of quantum (IOF letters) and classical (square shape) images are shown. The ratio between their mean intensities is stated on top of each image. In the middle row, the experimental results for our distillation technique through QHUL are presented. In the last row, a transverse cut of the distilled images is presented. We observe that, while the noise intensity increases, the phase estimation diminishes.

In the first experiment, while having superimposed classical and quantum images, QHUL is performed to distill the quantum image under different intensities of noise. We first characterized the signal flux rate emission measuring its mean intensity of an illuminated area on the camera. For the quantum image, *S*_0_ = 134 signal photons generated in a single pass through the crystal were used. The detection window was *T_D_* = 1 s, and the detection area was *S_D_* ≈ 32.5 μm by 32.5 μm. Signal intensity does not change during experiments. In a similar way but independently measured, different noise intensities are characterized, which are obtained by changing the angle of a linear polarizer in front of the laser in path *g*. The experiment starts superimposing the quantum and classical images on the camera. Experimental results are shown in Fig. 4. Its first row shows the superposition of classical and quantum images; noise intensity increases from left to right with the following ratios (*r* = mean signal intensity:mean noise intensity), *r* ≈ 1 : 8, *r* ≈ 1 : 37, *r* ≈ 1 : 50, *r* ≈ 1 : 104, and *r* ≈ 1 : 252. The second row in Fig. 4 shows distilled images by QHUL of the corresponding top superposed images. Imaging distillation through QHUL is successfully achieved in every case, even with a noise intensity 250 times higher than the signal intensity. However, we notice that, while noise intensity increases, sharpness of distilled images decreases. One can observe this in detail in the third row that shows a transverse cut of the distilled images (represented by a dotted red line). It is clear from our experimental results that phase accuracy diminishes as the noise intensity increases, which also corresponds to the prediction given in Eq. 8.

### Induced variance by noise

**Fig. 5.**
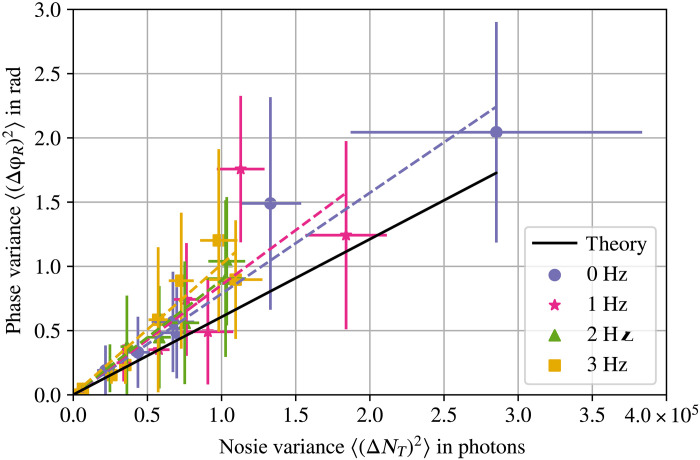
Distillation phase variance affected by noise variance. A light diffuser with four different rotation speeds is used to change the properties of the noise illumination; see supplementary text D. The different noise configurations are represented by different colors and symbols; see inset. Data points represent the experimental phase values obtained for different noise variances, and dashed lines represent their fits. A theoretical black solid line representing a Poissonian noise is also included. In all configurations, we observed that a higher noise variance increases the phase inaccuracy in QHUL. We also corroborate that the phase sensitivity is linearly dependent with the noise variance; for more details, see supplementary text F.

In a second experiment, we quantified the effects of noise variances on the phase accuracy of distilled images. The same configurations of noise intensities are used. In addition, a light diffuser mounted on a rotational motor with four angular frequencies of 0, 1, 2, and 3 Hz changed the noise variance. The noise variance is characterized considering the intensity variation of one pixel over 12 consecutive images. Experimental results are shown in Fig. 5. We plot the experimental measured values for the phase variance of distilled images against the noise variance for different angular frequencies (data point with error bars; purple circle, 0 Hz; rose star, 1 Hz; green triangle, 2 Hz; and yellow square, 3 Hz). We also provide a theoretical prediction for comparison (solid black line). The results show that an increment of the noise variance increases proportional with the phase variance. In addition, it can be observed that, in every case, a linear dependence appears between these two variances; see supplementary text F for more details. However, the noise variance introduces a slightly higher phase variance than expected. A reasonable explanation for this is that additional sources of noise were involved during the measurement process, such as airflow or temperature fluctuations. The experimental behavior of variances is in good agreement to theoretical predictions presented above in Eq. 8.

To conclude, we compare our distillation to other previously introduced techniques in Table 1. In our implementation, we have used at least five times more noise than in all previous related experiments, showing the highest resilience to date.

**Table 1.** Distillation performances.

Distillation techniques	Signal-to-noise ratio
*Phys. Rev. A* (*21*)	1:0.14
*Sci. Adv.* (*22*)	1:10
*Phys. Rev. A* (*23*)	1:49
*Sci. Adv.* (*24*)	1:5.8
*Sci. Rep.* (*25*)	1:20
Our work	1:252

## DISCUSSION

Our work explores the effects of noise in QIUL. We have also introduced a technique to distill the quantum image from that noise. Our quantum imaging distillation technique is based on QHUL (*35*). This technique uses a photon pair, signal and idler, where idler illuminates the object and signal is detected on the camera. The idler photon remains undetected, and its phase modulation is used in the imaging distillation procedure.

To prove our technique, we superimposed partially or completely a classical source of noise on top of our quantum image on the camera. Our technique worked in every occasion, even for noise intensities 250 times higher than our signal intensity. However, the noise variance does affect the phase accuracy of our distillation technique. A higher noise variance produces a higher phase inaccuracy, where, in general, these two quantities are linearly dependent.

**Fig. 6.**
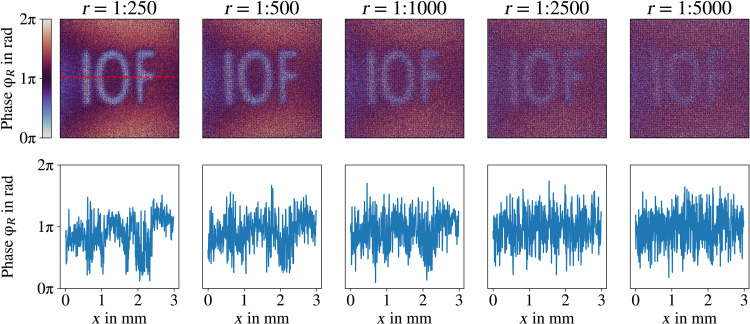
Simulated resilience limits. To find the limits of our technique, we have simulated a Poisonnian source of noise superimposed on our quantum image. The first row shows distilled images for different ratios stated above them. The second row shows a transverse cut of distilled images on top. The simulations show that our technique should be able to work at noise levels beyond 1000 times the quantum signal.

To extend our work and explore the limits of our technique, in Fig. 6, we present simulations of QHUL under extreme noise scenarios. For this, we have considered a noise with Poissonian statistics and with ratios up to 5000 times higher than the mean intensity of the signal of interest. The simulations show that our distillation technique keeps working until *r* = 1000 or even until *r* = 2500. For *r* = 5000, the distilled image is already blurred; however, it still exhibits some features of the original object.

Although, in our experiment, we used a classical source of noise, this distillation technique should also work for a quantum source of noise: for example, by replacing the noise laser with a phase-matched SPDC source and performing far field or near field imaging of the noisy object on the camera. Furthermore, because the underlying principle of this distillation technique is the phase modulation, our technique should be applicable to QIUL based on position correlations (*43*, *44*).

Our results are not just vital for QIUL but can have an important contribution to other techniques based on induced coherence without induced emission (*45*), such as spectroscopy (*46*), sensing (*47*), optical coherence tomography (*48*, *49*), entanglement certification (*50*), and quantum state tomography (*51*). Our experiment is a step forward for quantum imaging in open systems and could be useful to understand the limitations of a (quantum) Light Detection and Ranging (LIDAR) with undetected light.

## Supplementary Materials

### This PDF file includes:

Supplementary Materials and Methods Figs. S1 to S3 Table S1 References
