# Supplementary material for: Experimental quantum imaging distillation with undetected light
Source: Sci Adv. 2023 Aug 30;9(35):eadg9573. doi: 10.1126/sciadv.adg9573 (PMC10468131; doi:10.1126/sciadv.adg9573)
Supplement: Supplementary file 1 — Supplementary Materials and Methods Figs. S1 to S3 Table S1 References [file sciadv.adg9573_sm.pdf]

Science Advances
AAAS

Supplementary Materials for

Experimental quantum imaging distillation with undetected light

Jorge Fuenzalida *et al.*

Corresponding author: Jorge Fuenzalida, jorge.fuenzalida@tu-darmstadt.de; Juan P. Torres, juanp.torres@icfo.eu; Markus Gräfe, markus.graefe@tu-darmstadt.de

Sci. Adv. **9**, eadg9573 (2023)
DOI: 10.1126/sciadv.adg9573

This PDF file includes:

Supplementary Materials and Methods
Figs. S1 to S3
Table S1
References

A. The quantum state of signal photons.

In the undepleted pump beam approximation, if the bandwidth of parametric down-conversion (in the frequency and transverse wavenumber domains) is much larger than the corresponding bandwidth of the pump beam, the relationship between the quantum operators $a_S(\Omega, \mathbf{q})$ and $a_I(\Omega, \mathbf{q})$ (output face of the nonlinear crystal) and the operators $b_S(\Omega, \mathbf{q})$ and $b_I(\Omega, \mathbf{q})$ (input face of the nonlinear crystal) can be written in the low parametric gain approximation as (*52*)

$$
\begin{aligned}
a_S(\Omega, \mathbf{q}) =& b_S(\Omega, \mathbf{q}) \exp\left[i k_S(\Omega, \mathbf{q}) L \right] \\
&+ F_S(\Omega, \mathbf{q}) \int \mathrm{d}\omega_P \, \mathrm{d}\mathbf{q}_P \, E_P(\Omega_P, \mathbf{q}_P) \, b_I^\dagger(\Omega_P - \Omega, \mathbf{q}_P - \mathbf{q}), \\
a_I(\Omega, \mathbf{q}) =& b_I(\Omega, \mathbf{q}) \exp\left[i k_I(\Omega, \mathbf{q}) L \right] \\
&+ F_I(\Omega, \mathbf{q}) \int \mathrm{d}\omega_P \, \mathrm{d}\mathbf{q}_P \, E_P(\Omega_P, \mathbf{q}_P) \, b_S^\dagger(\Omega_P - \Omega, \mathbf{q}_P - \mathbf{q}),
\end{aligned} \tag{9}
$$

where

$$
F_{S,I}(\Omega, \mathbf{q}) = -i(\beta L) \, \mathrm{sinc}\left[\frac{\Delta_{S,I}(\Omega, \mathbf{q}) L}{2} \right] \exp\left\{ i \left[k_P^0 + k_{S,I}(\Omega, \mathbf{q}) - k_{I,S}(-\Omega, -\mathbf{q}) \right] \frac{L}{2} \right\}. \tag{10}
$$

The nonlinear coefficient β is

$$
\beta = \left(\frac{\hbar \, \omega_P \, \omega_S \, \omega_I \, [\chi^{(2)}]^2}{64 \, \pi^3 \, \epsilon_0 \, c^3 \, n_P \, n_S \, n_I} \right)^{1/2}, \tag{11}
$$

$n_{S,I,P}$ are refractive indexes, $\chi^{(2)}$ is the second-order nonlinear coefficient of the crystal, L is the crystal length, and $\omega_{P,S,I}$ are central frequencies. The phase mismatch functions are $\Delta_{S,I} = k_P^0 - k_{S,I}(\Omega, \mathbf{q}) - k_{I,S}(-\Omega, -\mathbf{q})$, $k_{S,I}$ are the wavenumbers of signal and idler waves, and k_P^0 is the wavenumber of the pump beam. The total number of photons N_P carried by the pump beam is

$$
N_P = \int \mathrm{d}\Omega \, \mathrm{d}\mathbf{q} \left| E_P(\Omega, \mathbf{q}) \right|^2. \tag{12}
$$

If we consider that signal and idler photons S_1 and I_1 (forward propagation modes) are re-injected back into the nonlinear crystal with the help of 4f systems, the operator associated to signal photon S_2 (backward propagation mode) is

$$a_{S_2}(\Omega,\mathbf{q}) = F_S(\Omega,\mathbf{q}) \Big\{ \exp\big[ik_S(\Omega,\mathbf{q})L + i\varphi_S(\Omega) + i\varphi_{P_1}\big]$$
$$+R^* \exp\big[-ik_I(-\Omega,-\mathbf{q})L + i\varphi_{P_2} - i\varphi_I(-\Omega)\big]\Big\} \int \mathrm{d}\omega_P\, \mathrm{d}\mathbf{q}_P\, E_P(\Omega_P,\mathbf{q}_P)\, b_I^\dagger(\Omega_P-\Omega,\mathbf{q}_P-\mathbf{q})$$
$$+F_S(\Omega,\mathbf{q}) \exp\big[i\varphi_{P_2}\big] \int \mathrm{d}\Omega_P\, \mathrm{d}\mathbf{q}_P\, E_P(\Omega_P,\mathbf{q}_P)\, f^\dagger(\Omega_P-\Omega,\mathbf{q}_P-\mathbf{q}). \quad (13)$$

Notice that in Eq. (13), for the sake of simplicity, we have omitted the terms that depend on the operator b_S since they will yield a zero contribution to the flux density of signal S_2 photons. We have made use of the approximation $k_I(\Omega_P-\Omega,\mathbf{q}_P-\mathbf{q}) \sim k_I(-\Omega,-\mathbf{q})$, since the bandwidth of the pump beam is assumed to be very small when compared with the bandwidth of parametric down-conversion. We also consider that the phases of the pump beams, φ_{P_1} and φ_{P_2}, that illuminate the nonlinear crystal might be different. The phases $\varphi_S(\Omega)$ and $\varphi_I(\Omega)$ are phases acquired by the signal S_1 and idler I_1 photons that traverse 4f systems before being re-injected back into the nonlinear crystal. The reflection coefficient of the object located in the idler I_1 path is $R = |R|\exp(i\varphi_R)$. The operator f takes into account (*53*) the presence of the object with reflectivity R. These operators fulfill the relationships $\Big[f(\Omega,\mathbf{q}), f^\dagger(\Omega',\mathbf{q}')\Big] = \big(1-|R|^2\big)\,\delta(\Omega-\Omega')\,\delta(\mathbf{q}-\mathbf{q}')$.

B. Flux density of signal photons detected in a 2f system

We detect the flux density of signal photons S_2 with the help of a 2f system with focal length f. The input-output relationship between operators is similar to the classical relationship (*54*)

$$a_{S_2}(t,\mathbf{x}_2) = \frac{1}{\lambda_S f} \int \mathrm{d}\mathbf{x}_1\, a_{S_2}(t,\mathbf{x}_1) \exp\left[-i\frac{2\pi}{\lambda_S f}\mathbf{x}_1\cdot\mathbf{x}_2\right], \quad (14)$$

where $\mathbf{x}_1$ and $\mathbf{x}_2$ are the corresponding transverse coordinates in the input and output planes of the 2f system. For the sake of simplicity, we ignore any global phase. If we introduce the Fourier transform of the operator $a_{S_2}(t,\mathbf{x}_1)$ as $a_{S_2}(t,\mathbf{x}) = (2\pi)^{-3/2} \int \mathrm{d}\Omega\, \mathrm{d}\mathbf{q}\, a_{S_2}(\Omega,\mathbf{q})\, \exp\Big[i\mathbf{q}\cdot\mathbf{x} - i\Omega t\Big]$, we can write Eq. (14) as

$$a_{S_2}(t,\mathbf{x}_2) = \frac{(2\pi)^{1/2}}{\lambda_S\, f} \int \mathrm{d}\Omega\, a_{S_2}\left(\Omega, \frac{2\pi}{\lambda_S\, f}\mathbf{x}_2\right)\, \exp\left(-i\,\Omega\, t\right), \tag{15}$$

where we have made use of the identity $\int \mathrm{d}\mathbf{x}\, \exp\Big[i\big(\mathbf{q}-\mathbf{q}'\big)\cdot\mathbf{x}\Big] = (2\pi)^2\delta(\mathbf{q}-\mathbf{q}')$. The flux density (photons/m^2) of signal photons detected at position $\mathbf{x}_2$ is

$$N(\mathbf{x}_2) = \frac{(2\pi)^2}{(\lambda_S f)^2}\int \mathrm{d}\Omega\, \left\langle a_S^\dagger\left(\Omega, \frac{2\pi}{\lambda_S f}\mathbf{x}_2\right)\, a_S\left(\Omega, \frac{2\pi}{\lambda_S f}\mathbf{x}_2\right)\right\rangle. \tag{16}$$

If we make use of Eq. (13) into Eq. (16) we obtain

$$N(\mathbf{x}_2) = \frac{(2\pi)^2}{(\lambda_S f)^2}\, N_p \int \mathrm{d}\Omega\, \left|F_S\left(\Omega, \frac{2\pi}{\lambda_S f}\mathbf{x}_2\right)\right|^2 \Big\{1 - |R|^2 + \Big|\exp\big[ik_S(\Omega,\mathbf{q})L + i\varphi_S(\Omega)\big] + R^*\, \exp\big[-ik_I(-\Omega,-\mathbf{q})L - i\varphi_I(-\Omega)\big]\Big|^2\Big\}, \tag{17}$$

where $N_P = \mathcal{I}_P S_P T_P$. $\mathcal{I}_P$ is the peak intensity of the pump beam, S_P its area, and T_P its time duration. If we make the substitution $\beta^2 = \sigma/[(2\pi)^3\mathcal{I}_P]$, define $V_S(\Omega) \equiv V_S(\Omega,\mathbf{x}_2 = 0)$, and associate a large value of T_P with the detection time T_D, the number of photons detected in a small area S_D centered around $\mathbf{x}_2 = 0$ is

$$N_0 = \frac{S_P S_D}{(\lambda_S f)^2}\,\frac{T_D}{\pi}\int \mathrm{d}\Omega\, \Big|V_S(\Omega)\Big|^2 \Big\{1 + |R|\cos(\theta + \varphi_R)\Big\}. \tag{18}$$

V_S is the same functions as F_S in Eq. (10) by substituting the nonlinear coefficient β by σ. We should notice that similar expressions to Eq. (18) for describing flux rates with detection systems based on 2f systems has been derived using other methods (*55, 56*).

If we approximate the phase matching function as $\Delta_S = D\Omega$, where D is the difference of inverse group velocities at the central frequencies between signal and idler photons,

$$\theta = k_P^0 L + \frac{\omega_S L_S + \omega_I L_I}{c} + \Omega\left[DL + \frac{L_S - L_I}{c}\right] + \varphi_{P_1} - \varphi_{P_2}. \tag{19}$$

$L_{S,I}$ are the lengths of the 4f systems traversed by signal and idler photons, respectively, before being re-injected back into the nonlinear crystal. For the sake of simplicity, we write

$$|V(\Omega)|^2 = \exp\left(-\pi \frac{\Omega^2}{B^2}\right). \tag{20}$$

where B is the bandwidth of parametric down-conversion. Making the integration over frequency in Eq. (18), we obtain

$$\langle N_0 \rangle = 2S_0 \left[1 + |R|\gamma \cos(\delta + \varphi_R)\right], \tag{21}$$

with $\delta = k_P^0 L + (\omega_S L_S + \omega_I L_I)/c$,

$$S_0 = \frac{S_P\, S_D}{(\lambda_S f)^2} \frac{T_D B}{2\pi} (\sigma L)^2, \tag{22}$$

and

$$\gamma = \exp\left[-\frac{B^2}{4\pi}\left(DL + \frac{L_s - L_i}{c}\right)^2\right]. \tag{23}$$

Notice that the visibility of signal $\langle N_0 \rangle$ as function of phase δ is

$$V = |R|\gamma. \tag{24}$$

N_0 is the variable that we designate as N_S in the main text.

C. Sensitivity of phase estimation with phase shifting digital holography under the presence of external noise

For the sake of simplicity, we write below $N_S = N_0 \equiv N$. We aim at estimating φ_R using M phases ($\delta_j = j\, 2\pi/M$ with $j = 0, 1, ..., M-1$). The expression for the estimation of the phase is

$$\varphi_R = -\tan^{-1} \frac{\sum_j \langle N \rangle_j \sin(\delta_j)}{\sum_j \langle N \rangle_j \cos(\delta_j)}. \tag{25}$$

For M=4 (phases $\delta_j = 0, \pi/2, \pi, 3\pi/2$) we have

$$\varphi_R = \tan^{-1} \frac{\langle N \rangle_{3\pi/2} - \langle N \rangle_{\pi/2}}{\langle N \rangle_0 - \langle N \rangle_\pi}. \tag{26}$$

We will make use of the error propagation formula

$$\langle (\Delta\varphi_R)^2 \rangle = \sum_j \left(\frac{\partial \varphi_R}{\partial \langle N \rangle_j} \right)^2 \left[\langle (\Delta N)^2 \rangle_j + \left\langle (\Delta N_T)^2 \right\rangle \right]. \tag{27}$$

where $\langle N_T \rangle$ is the background signal (independent of the signal of interest) that reaches the detector with a variance of $\langle (\Delta N_T)^2 \rangle$. If the signal N_j has a bandwidth B, and the detection time T_D is large, i.e., $T_D \gg 1/B$, a condition that applies in the experiment, we can safely write that $\langle (\Delta N)^2 \rangle_j = \langle N \rangle_j$. Therefore, we can write

$$\langle (\Delta\varphi_R)^2 \rangle = \sum_j \left(\frac{\partial \varphi_R}{\partial \langle N \rangle_j} \right)^2 \left[\langle N \rangle_j + \left\langle (\Delta N_T)^2 \right\rangle \right]. \tag{28}$$

The derivatives are

$$\frac{\partial \varphi_R}{\partial \langle N \rangle_i} = \frac{\sum_j \langle N \rangle_j \sin\delta_j \cos\delta_i - \sum_j \langle N \rangle_j \cos\delta_j \sin\delta_i}{\left[\sum_j \langle N \rangle_j \sin\delta_j \right]^2 + \left[\sum_j \langle N \rangle_j \cos\delta_j \right]^2}. \tag{29}$$

After some calculations, we obtain

$$\begin{aligned} \sum_j \langle N \rangle_j \sin(\delta_j) &= -M|R|\gamma\, S_0 \sin(\varphi_R), \\ \sum_j \langle N \rangle_j \cos(\delta_j) &= M|R|\gamma\, S_0 \cos(\varphi_R), \end{aligned} \tag{30}$$

where we have made use of

$$\sum_i \sin^2(\varphi_R + \delta_j) = \frac{M}{2}, \tag{31}$$

and

$$\sum_j \sin^2(\varphi_R - \delta_j) \cos(\varphi_R + \delta_j) = 0. \tag{32}$$

We can easily verify numerically the validity of these expressions for several values of M. Finally, we obtain

$$\left(\frac{\partial \varphi_R}{\partial \langle N \rangle_j}\right)^2 = \frac{1}{M^2\gamma^2|R|^2}\frac{1}{S_0^2}\,\sin^2(\varphi_R+\delta_j). \tag{33}$$

The sensitivity is

$$\begin{aligned}\langle(\Delta\varphi_R)^2\rangle &= \sum_j \left(\frac{\partial \varphi_R}{\partial \langle N \rangle_j}\right)^2 \left[\langle N\rangle_j + \left\langle(\Delta N_T)^2\right\rangle\right] = \frac{1}{M^2\gamma^2|R|^2}\frac{1}{S_0^2}\\ &\times\Big\{2S_0\sum_j \sin^2(\varphi_R+\delta_j) + 2|R|\gamma\, S_0\sum_j \sin^2(\varphi_R+\delta_j)\,\cos(\varphi_R+\delta_j)\\ &+\left\langle(\Delta N_T)^2\right\rangle\sum_j \sin^2(\varphi_R+\delta_j)\Big\} = \frac{1}{M^2\gamma^2|R|^2}\frac{1}{S_0^2}M\left\{S_0 + \frac{\langle(\Delta N_T)^2\rangle}{2}\right\}.\end{aligned} \tag{34}$$

If we make the measurement n times, the phase sensitivity is

$$\langle(\Delta\varphi_R)^2\rangle = \frac{1}{M\gamma^2|R|^2\,n\,S_0}\left[1+\frac{\langle(\Delta N_T)^2\rangle}{2S_0}\right]. \tag{35}$$

If we make use of the visibility V given by Eq. (24), we can write Eq. (35) as

$$\langle(\Delta\varphi_R)^2\rangle = \frac{1}{MV^2\,n\,S_0}\left[1+\frac{\langle(\Delta N_T)^2\rangle}{2S_0}\right]. \tag{36}$$

D. Characterization of the noise

We characterized the intensity and variance of the noise. In front of the noise source, we placed a linear polarizer to vary its intensity. We also placed a light diffuser in the path to the camera that rotates at different angular frequencies. In Fig. S1, we plot experimental data for the noise variances against noise intensities. The purple circle represents an angular frequency of 0 Hz. Similarly, the pink start, green triangle, and yellow square represent angular frequencies of 1, 2, and 3 Hz. A theoretical black line representing $\langle(\Delta N_T)^2\rangle = \langle N_T\rangle$, is also provided. All configurations of noise employed in the experiments show super-Poissonian statistics.

E. Signal intensity affected by noise

In quantum holography with undetected light (QHUL), the signal intensity varies depending on the phase value. If an additional source of noise is superimposed on the camera, the signal variance increases. Figure S2 shows five QHUL measurements of 12 steps for the signal intensity collected by one pixel. We have also superimposed a noise in different ratios to the mean signal intensity. Solid-shaded areas represent the obtained signal variances. The blue area represents a ratio of $r \approx 1 : 8$ and resulted in a small signal variance. The orange area represents a ratio of $r \approx 1 : 91$ and shows an increment in the signal variance with respect to the blue one. Finally, the green area represents a ratio of $r \approx 1 : 252$, and we obtained the biggest variance for the signal photon.

F. Statistical analysis of results presented in Fig. 5

In this appendix we present a further statistical analysis of the data presented in Fig. 5. These values can be found in Table S1. For our statistical analysis we employ the so-called *log-log plot* which is a technique well-known in optical coherence tomography (*57*). By this we obtain a linear function $Y = mX + b$, where $X = \log\{\langle(\Delta N_T)^2\rangle\}$ and $Y = \log\{\langle(\Delta\varphi_R)^2\rangle\}$. m is the angular coefficient that for $m = 1$ represents a linear function. In Fig. S3 are presented the fit curves, obtaining all m-values close to 1. This confirms that the noise and phase variances have a linear dependence.

Tables

	Noise variance $\langle(\Delta N_T)^2\rangle$	Phase variance $\langle(\Delta\varphi_R)^2\rangle$
0 Hz	0	0.011 ± 0.005
	6292±1042	0.044±0.025
	21728±5609	0.191±0.194
	43617±7997	0.331±0.277
	67202±14342	0.568±0.390
	69767±10895	0.483±0.355
	132928±20766	1.490±0.827
	285295± 98281	2.044±0.858
1 Hz	0	0.011 ± 0.005
	6498±1135	0.045 ±0.024
	33989±4785	0.248±0.144
	57801±7108	0.351±0.297
	76327±8422	0.743±0.439
	90763±17137	0.491±0.409
	112891±16084	1.757±0.570
	183957±27457	1.243±0.733
2 Hz	0	0.011 ± 0.005
	6364±720	0.047±0.024
	24719±3254	0.207±0.186
	36157±4295	0.375±0.397
	57995±7749	0.448±0.399
	75174±9738	0.560± 0.477
	102718±13822	0.905±0.609
	103500± 12408	1.040±0.499
3 Hz	0	0.011 ± 0.005
	6511±926	0.047±0.023
	25715±4189	0.154±0.067
	35132±4263	0.232±0.146
	57250±6479	0.584±0.565
	72825±8410	0.888±0.529
	98111±12569	1.202±0.711
	109504±18029	0.897±0.462

Table S1: **Detailed results presented in Fig. 5.** The noise variance errors correspond to the different values obtained at different camera pixels. The quantum holography procedure augments some errors in the phase variance because they have eluded the unwrapping script.

Figures

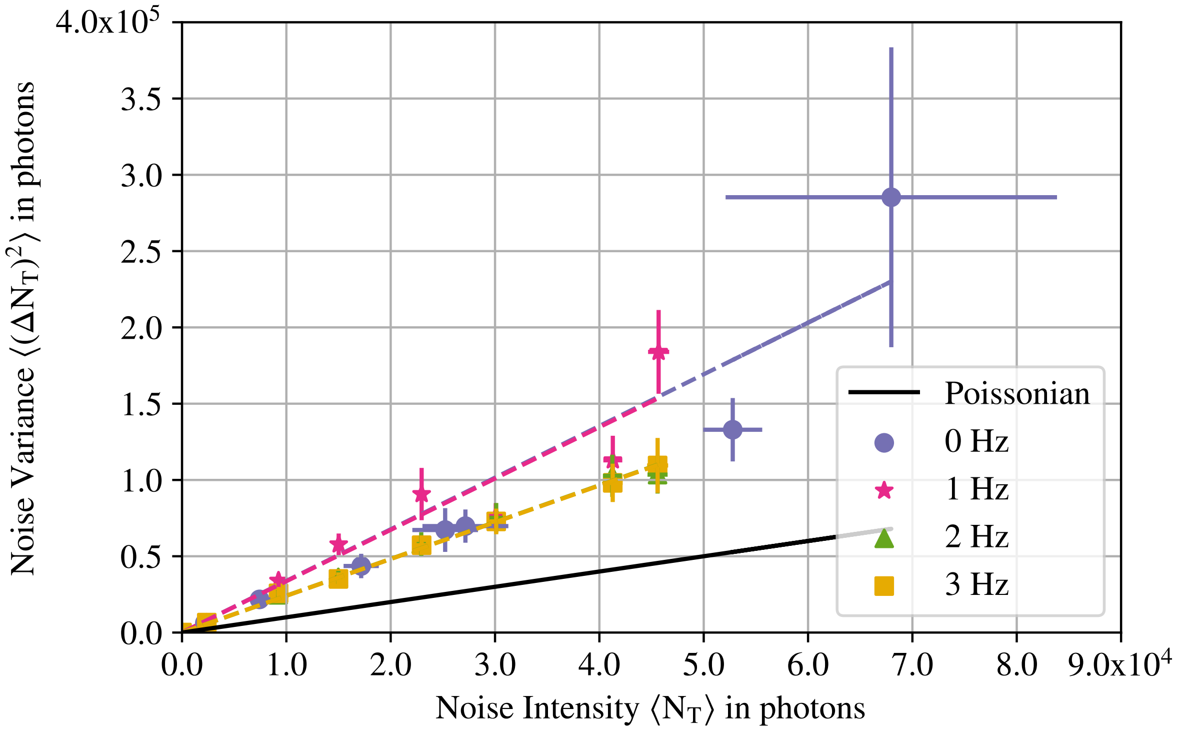

Figure S1: **Noise variance against noise intensity.** The noise properties were characterized for different configurations. For this, the mean intensity of the noise was increased while a light diffuser was rotating at different speeds. Experimental data points of the noise variances are plotted against the noise intensities. The detection window was set to be $T_D = 1$ s and the detection area S_D was $32.5 \times 32.5\ \mu\text{m}^2$. A theoretical black line represents the Poissonian case, e.g., $\langle(\Delta N_T)^2\rangle = \langle N_T\rangle$. In all the experiments, the noise exhibits super-Poissonian statistics.

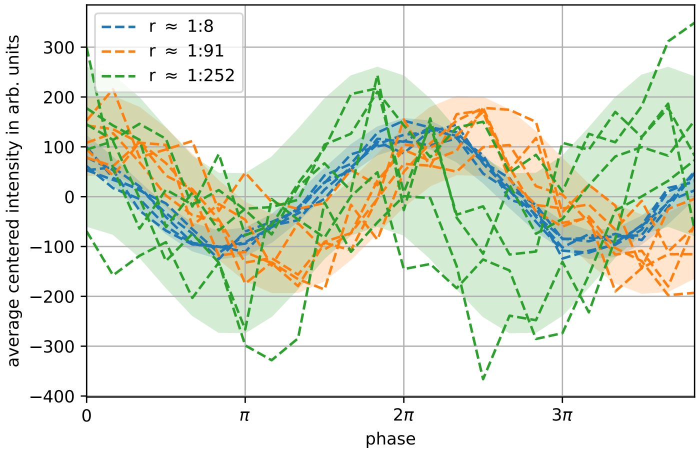

Figure S2: **Signal intensity in QHUL affected by noise.** Applying a QHUL of 12 steps, we obtained the signal intensity against the phase. The experimental signal intensities are shown with dotted lines. The solid-shaded areas show the signal variances for different noise intensities. A higher noise intensity increases the signal variance. More details in the main text.

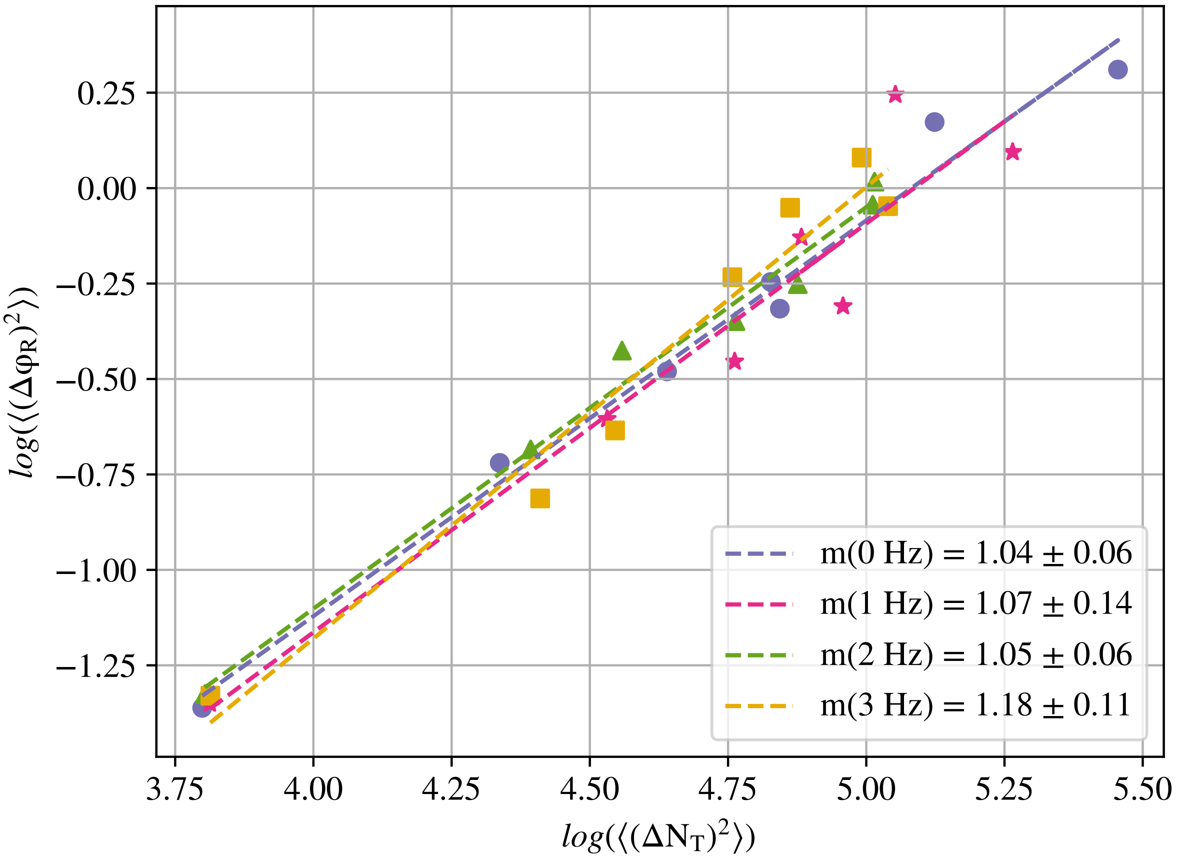

Figure S3: **Statistical analysis on the noise and phase variances**. The log-log values of the experimental data are represented by different shapes and colors for different angular speeds, see inset. A linear function $Y = mX + b$ is fit over the data points, where m is the angular coefficient. By having values of m near to 1 for all fit curves, we can confirm that the noise and phase variances have a linear dependence between them.

REFERENCES AND NOTES

1. M. Gilaberte Basset, F. Setzpfandt, F. Steinlechner, E. Beckert, T. Pertsch, M. Gräfe, Perspectives for applications of quantum imaging. *Laser Photonics Rev.* **13**, 1900097 (2019).

2. R. S. Aspden, D. S. Tasca, R. W. Boyd, M. J. Padgett, EPR-based ghost imaging using a single-photon-sensitive camera. *New J. Phys.* **15**, 073032 (2013).

3. P. A. Morris, R. S. Aspden, J. E. C. Bell, R. W. Boyd, M. J. Padgett, Imaging with a small number of photons. *Nat. Commun.* **6**, 5913 (2015).

4. G. B. Lemos, V. Borish, G. D. Cole, S. Ramelow, R. Lapkiewicz, A. Zeilinger, Quantum imaging with undetected photons. *Nature* **512**, 409–412 (2014).

5. M. Gilaberte Basset, A. Hochrainer, S. Töpfer, F. Riexinger, P. Bickert, J. R. Leòn-Torres, F. Steinlechner, M. Gräfe, Video-rate imaging with undetected photons. *Laser Photonics Rev.* **15**, 2000327 (2021).

6. O. Schwartz, J. M. Levitt, R. Tenne, S. Itzhakov, Z. Deutsch, D. Oron, Superresolution microscopy with quantum emitters. *Nano Lett.* **13**, 5832–5836 (2013).

7. A. Classen, J. von Zanthier, M. O. Scully, G. S. Agarwal, Superresolution via structured illumination quantum correlation microscopy. *Optica* **4**, 580–587 (2017).

8. M. Unternährer, B. Bessire, L. Gasparini, M. Perenzoni, A. Stefanov, Super-resolution quantum imaging at the Heisenberg limit. *Optica* **5**, 1150 (2018).

9. R. Tenne, U. Rossman, B. Rephael, Y. Israel, A. Krupinski-Ptaszek, R. Lapkiewicz, Y. Silberberg, D. Oron, Super-resolution enhancement by quantum image scanning microscopy. *Nat. Photon.* **13**, 116–122 (2019).

10. G. Brida, M. Genovese, I. R. Berchera, Experimental realization of sub-shot-noise quantum imaging. *Nat. Photon.* **4**, 227–230 (2010).

11. J. Sabines-Chesterking, A. McMillan, P. Moreau, S. Joshi, S. Knauer, E. Johnston, J. Rarity, J. Matthews, Twin-beam sub-shot-noise raster-scanning microscope. *Opt. Express* **27**, 30810–30818 (2019).

12. C. A. Casacio, L. S. Madsen, A. Terrasson, M. Waleed, K. Barnscheidt, B. Hage, M. A. Taylor, W. P. Bowen, Quantum-enhanced nonlinear microscopy. *Nature* **594**, 201–206 (2021).

13. M. C. Teich, B. E. A. Saleh, Entangled-photon microscopy. *Cesk. Cas. Fyz* **47**, 3–8 (1997).

14. B. Ndagano, H. Defienne, D. Branford, Y. D. Shah, A. Lyons, N. Westerberg, E. M. Gauger, D. Faccio, Quantum microscopy based on Hong–Ou–Mandel interference. *Nat. Photon.* **16**, 384–389 (2022).

15. H. Defienne, B. Ndagano, A. Lyons, D. Faccio, Polarization entanglement-enabled quantum holography. *Nat. Phys.* **17**, 591–597 (2021).

16. R. Camphausen, Á. Cuevas, L. Duempelmann, R. A. Terborg, E.Wajs, S. Tisa, A. Ruggeri, I. Cusini, F. Steinlechner, V. Pruneri, A quantum-enhanced wide-field phase imager. *Sci. Adv.* **7**, eabj2155 (2021).

17. S. Lloyd, Enhanced sensitivity of photodetection via quantum illumination. *Science* **321**, 1463–1465 (2008).

18. S.-H. Tan, B. I. Erkmen, V. Giovannetti, S. Guha, S. Lloyd, L. Maccone, S. Pirandola, J. H. Shapiro, Quantum illumination with Gaussian states. *Phys. Rev. Lett.* **101**, 253601 (2008).

19. E. Lopaeva, I. R. Berchera, I. P. Degiovanni, S. Olivares, G. Brida, M. Genovese, Experimental realization of quantum illumination. *Phys. Rev. Lett.* **110**, 153603 (2013).

20. J.-W. Pan, C. Simon, Č. Brukner, A. Zeilinger, Entanglement purification for quantum communication. *Nature* **410**, 1067–1070 (2001).

21. D. G. England, B. Balaji, B. J. Sussman, Quantum-enhanced standoff detection using correlated photon pairs. *Phys. Rev. A* **99**, 023828 (2019).

22. H. Defienne, M. Reichert, J. W. Fleischer, D. Faccio, Quantum image distillation. *Sci. Adv.* **5**, eaax0307 (2019).

23. Y. Zhang, D. England, A. Nomerotski, P. Svihra, S. Ferrante, P. Hockett, B. Sussman, Multidimensional quantum-enhanced target detection via spectrotemporal-correlation measurements. *Phys. Rev. A* **101**, 053808 (2020).

24. T. Gregory, P. A. Moreau, E. Toninelli, M. J. Padgett, Imaging through noise with quantum illumination. *Sci. Adv.* **6**, eaay2652 (2020).

25. T. Gregory, P. A. Moreau, S. Mekhail, O. Wolley, M. J. Padgett, Noise rejection through an improved quantum illumination protocol. *Sci. Rep.* **11**, 21841 (2021).

26. J. Zhao, A. Lyons, A. C. Ulku, H. Defienne, D. Faccio, E. Charbon, Light detection and ranging with entangled photons. *Opt. Express* **30**, 3675–3683 (2022).

27. M. Lahiri, R. Lapkiewicz, G. B. Lemos, A. Zeilinger, Theory of quantum imaging with undetected photons. *Phys. Rev. A* **92**, 013832 (2015).

28. J. Fuenzalida, A. Hochrainer, G. B. Lemos, E. A. Ortega, R. Lapkiewicz, M. Lahiri, A. Zeilinger, Resolution of quantum imaging with undetected photons. *Quantum* **6**, 646 (2022).

29. A. Vega, A. Santos, J. Fuenzalida, M. Gilaberte Basset, T. Pertsch, M. Gräfe, S. Saravi, F. Setzpfandt, Fundamental resolution limit of quantum imaging with undetected photons. *Phys. Rev. Res.* **4**, 033252 (2022).
30. I. Kviatkovsky, H. M. Chrzanowski, E. G. Avery, H. Bartolomaeus, S. Ramelow, Microscopy with undetected photons in the mid-infrared. *Sci. Adv.* **6**, eabd0264 (2020).
31. A. V. Paterova, S. M. Maniam, H. Yang, G. Grenci, L. A. Krivitsky, Hyperspectral infrared microscopy with visible light. *Sci. Adv.* **6**, eabd0460 (2020).
32. A. V. Paterova, H. Yang, Z. S. D. Toa, L. A. Krivitsky, Quantum imaging for the semiconductor industry. *Appl. Phys. Lett.* **117**, 054004 (2020).
33. A. V. Paterova, D. A. Kalashnikov, E. Khaidarov, H. Yang, T. W. W. Mass, R. Paniagua-Domínguez, A. I. Kuznetsov, L. A. Krivitsky, Non-linear interferometry with infrared metasurfaces. *Nanophotonics* **10**, 1775–1784 (2021).
34. B. E. Haase, J. Hennig, M. Kutas, E. Waller, J. Hering, G. von Freymann, D. Molter, Phase-quadrature quantum imaging with undetected photons. *Opt. Express* **31**, 143–152 (2023).
35. S. Töpfer, M. Gilaberte Basset, J. Fuenzalida, F. Steinlechner, J. P. Torres, M. Gräfe, Quantum holography with undetected light. *Sci. Adv.* **8**, eabl4301 (2022).
36. D. Klyshko, Scattering of light in a medium with nonlinear polarizability. *Sov. Phys. JETP* **28**, 522 (1969).
37. D. C. Burnham, D. L. Weinberg, Observation of simultaneity in parametric production of optical photon pairs. *Phys. Rev. Lett.* **25**, 84–87 (1970).
38. S. P. Walborn, C. Monken, S. Pádua, P. S. Ribeiro, Spatial correlations in parametric down-conversion. *Phys. Rep.* **495**, 87–139 (2010).
39. M. Lahiri, A. Hochrainer, R. Lapkiewicz, G. B. Lemos, A. Zeilinger, Nonclassicality of induced coherence without induced emission. *Phys. Rev. A* **100**, 053839 (2019).
40. H. Wiseman, K. Mølmer, Induced coherence with and without induced emission. *Phys. Lett. A* **270**, 245–248 (2000).
41. B. Yurke, S. L. McCall, J. R. Klauder, SU(2) and SU(1,1) interferometers. *Phys. Rev. A* **33**, 4033–4054 (1986).
42. X. Y. Zou, L. J. Wang, L. Mandel, Induced coherence and indistinguishability in optical interference. *Phys. Rev. Lett.* **67**, 318–321 (1991).

43. B. Viswanathan, G. B. Lemos, M. Lahiri, Resolution limit in quantum imaging with undetected photons using position correlations. *Opt. Express* **29**, 38185–38198 (2021).
44. I. Kviatkovsky, H. M. Chrzanowski, S. Ramelow, Mid-infrared microscopy via position correlations of undetected photons, *Opt. Express* **30**, 5916–5925 (2022).
45. A. Hochrainer, M. Lahiri, M. Erhard, M. Krenn, A. Zeilinger, Quantum indistinguishability by path identity and with undetected photons. *Rev. Mod. Phys.* **94**, 025007 (2022).
46. D. A. Kalashnikov, A. V. Paterova, S. P. Kulik, L. A. Krivitsky, Infrared spectroscopy with visible light. *Nat. Photon.* **10**, 98–101 (2016).
47. M. Kutas, B. Haase, P. Bickert, F. Riexinger, D. Molter, G. von Freymann, Terahertz quantum sensing. *Sci. Adv.* **6**, eaaz8065 (2020).
48. A. Vallés, G. Jiménez, L. J. Salazar-Serrano, J. P. Torres, Optical sectioning in induced coherence tomography with frequency-entangled photons. *Phys. Rev. A* **97**, 023824 (2018).
49. A. V. Paterova, H. Yang, C. An, D. A. Kalashnikov, L. A. Krivitsky, Tunable optical coherence tomography in the infrared range using visible photons. *Quantum Sci.Technol.* **3**, 025008 (2018).
50. G. B. Lemos, R. Lapkiewicz, A. Hochrainer, M. Lahiri, A. Zeilinger, One-photon measurement of two-photon entanglement. *Phys. Rev. Lett.* **130**, 090202 (2023).
51. J. Fuenzalida, J. Kysela, K. Dovzhik, G. B. Lemos, A. Hochrainer, M. Lahiri, A. Zeilinger, Quantum state tomography of undetected photons. arXiv: 2211.10304 (2022).
52. B. Dayan, Theory of two-photon interactions with broadband down-converted light and entangled photons. *Phys. Rev. A* **76**, 043813 (2007).
53. R. W. Boyd, G. S. Agarwal, K. W. C. Chan, A. K. Jha, M. N. O'Sullivan, Propagation of quantum states of light through absorbing and amplifying media. *Opt. Commun.* **281**, 3732–3738 (2008).
54. J. W. Goodman, *Introduction to Fourier Optics* (Roberts and Company Publishers, 2005).
55. E. Brambilla, A. Gatti, L. Lugiato, M. Kolobov, Quantum structures in traveling-wave spontaneous parametric down-conversion. *Eur. Phys. J. D* **15**, 127–135 (2001).
56. E. Brambilla, A. Gatti, M. Bache, L. A. Lugiato, Simultaneous near-field and farfield spatial quantum correlations in the high-gain regime of parametric down-conversion. *Phys. Rev. A* **69**, 023802 (2004).
57. C. Vinegoni, J. S. Bredfeldt, D. L. Marks, S. A. Boppart, Nonlinear optical contrast enhancement for optical coherence tomography. *Opt. Express* **12**, 331–341 (2004).
